# Evaluation of the accuracy of renal depth estimation formulas in horseshoe kidney

**DOI:** 10.1097/MD.0000000000009141

**Published:** 2017-12-08

**Authors:** Guangyu Ma, Yingmao Chen, Mingzhe Shao, Jiahe Tian, Baixuan Xu

**Affiliations:** Department of Nuclear Medicine, Chinese PLA General Hospital, Beijing, China.

**Keywords:** glomerular filtration rate, horseshoe kidney, radionuclide renography, renal depth

## Abstract

Estimation formulas are usually used to calculate renal depth when glomerular filtration rate (GFR) is measured by the Gates method. Horseshoe kidney (HSK) anatomical structure is different from the normal form of the kidney. The existing formulas are based on the normal form. It is unknown whether the existing formulas are valid in HSK patients. This study was performed to estimate the accuracy of the existing 6 renal depth estimation formulas in HSK.

Renal depth and total thickness (T, cm) of the body at the level of the kidneys were measured by CT in 94 HSK patients. Their sex, age, height (H, cm), and weight (W, kg) were recorded. The existing 6 estimation formulas were used to obtain estimated renal depth. Correlation coefficients, Bland-Altman analysis, and paired *t* test were performed between estimated and the CT measured renal depth.

Estimated renal depths were all lower than the CT measured renal depths and there was significant difference between estimated and CT measured renal depth. The CT measured renal depth and estimated renal depth derived from Ma GY formula correlated best (right: *r* = 0.80, *P* < .01; left: *r* = 0.77, *P* < .01). The renal depth derived from Tonnesen formula was significantly lower than the CT measured renal depth. The agreement between the estimated renal depth derived from Tonnesen formula and the CT measured renal depth was the worst, with the mean difference of (right: −3.11 ± 1.13 cm; left: −2.79 ± 1.07 cm). The agreement between the estimated renal depth derived from Li Q formula and Ma GY formula and the CT measured renal depth was the best, with the mean difference of right: −1.68 ± 1.09 cm; left: −1.32 ± 1.06 cm and right: −1.59 ± 1.01 cm; left: −1.59 ± 0.99 cm, respectively. But the greatest error of the difference between Li Q formula and Ma GY formula estimated depth and the CT measured depth was up to −4.83 cm, and the estimated deviation is unacceptable.

All the existing formulas do not fully apply to HSK. To provide reliable and accurate estimates of renal depth, we should develop a new formula to estimate the renal depth in HSK patients.

## 1 Introduction

Horseshoe kidney (HSK), a congenital anomaly of renal fusion, is one of the most common renal anomalies.^[1]^ For HSK patients and patients with kidney diseases, it is important to accurately evaluate renal function to determine a suitable treatment plan.^[2]^ Accurate assessment of glomerular filtration rate (GFR) is essential for interpreting clinical symptoms, drug dosing, detecting and managing kidney disease, and assessing prognosis.^[3]^ GFR refers to the amount of ultra-filtrate kidneys generated per unit time, which is an important indicator of kidney function.^[4]^

Renal dynamic imaging with Tc-99m DTPA (diethylenetriaminepentaacetic acid) is an ideal method for the determination of GFR also known as the Gates method.^[5]^ However, the accuracy of this method is affected by many factors; among them, renal depth is an important one. Renal depth deviation can cause GFR error,^[6]^ a +/− 1 cm error in true kidney depth which may cause a 18% difference in GFR in adults.^[4]^

Estimation formulas are often used to calculate renal depth. The existing 6 formulas^[4,7–11]^ are based on the normal form of the kidney. Most HSK patients have abnormal kidney rotation and fusion of the kidneys at the lower poles to form an isthmus, and its anatomical structure is different from the normal form. It is unknown whether the existing formulas are valid in HSK patients. In this study, we therefore examined the accuracy of the existing 6 renal depth estimation formulas in HSK patients.

## 2 Materials and methods

### 2.1 Materials

**Figure 1:**
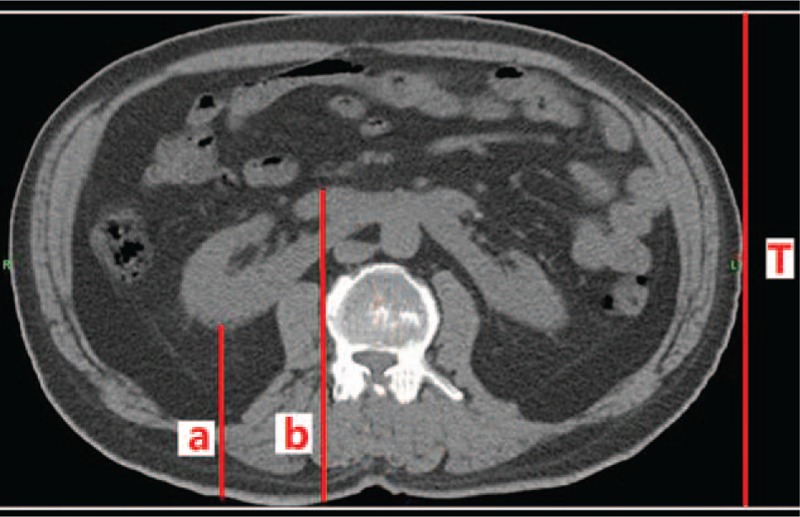
CT scan showing skin to anterior and posterior renal surfaces at the level of the renal hilum. Renal depth was determined by averaging the anterior and posterior depths at the renal hilum: renal depth = (a + b)/2; T is total thickness of the body at the level of the kidneys.

The study was approved by the Ethic Committee of Chinese PLA General Hospital and the written informed consent was obtained from each patient. The research objects of this article were patients undergoing routine clinical PET/CT or CT studies, and 94 HSK patents were selected. Patients with ascites, a single kidney, or masses that might distort the renal depth were excluded. Renal depth was determined by measuring from the skin on the posterior aspect of the renal at the renal hilum and then taking an average of these values to determine a mean depth (Fig. 1). The total thickness (T, cm) of the body at the level of the kidneys was also measured by CT (Fig. 1). The following data were recorded: sex, age, height, weight, thickness, and renal depth (Table 1).

**Table 1:**
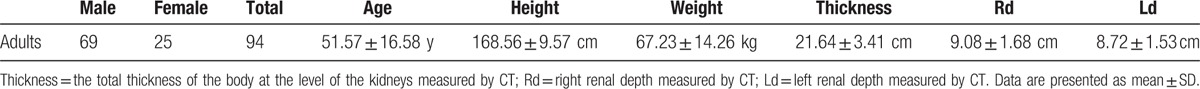
The general information of 94 HSK patents.

### 2.2 Methods

Estimation formulas were used to obtain estimated renal depth. Estimated renal depth was compared with the CT measured renal depth.

The existing 6 formulas are as follows:

1. Tonnesen formula^[7]^: 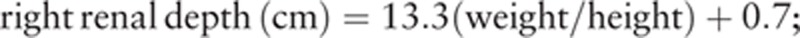 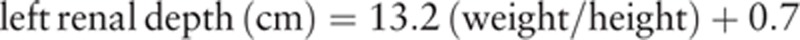
2. Taylor formula^[8]^: 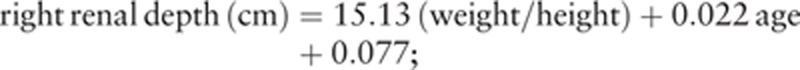 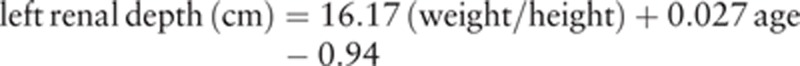
3. Inoue formula^[9]^: 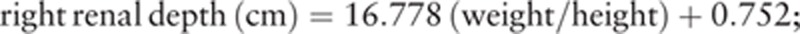 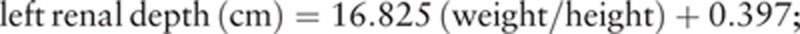
4. Li Q formula^[10]^: 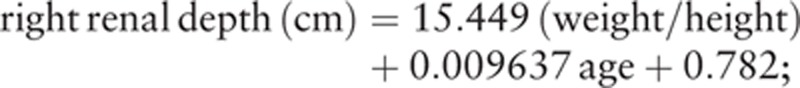 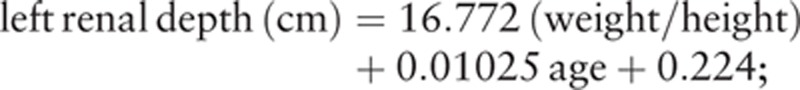
5. Ma GY formula^[4]^: 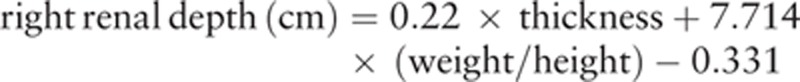 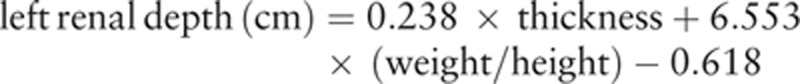
6. Xue JJ formula^[11]^: 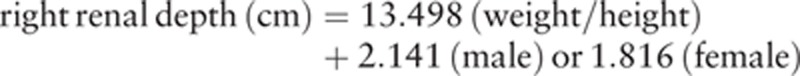 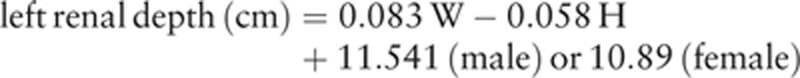

### 2.3 Statistical analysis

All data were expressed as mean ± standard deviation of the mean (SD). Correlation analysis was performed between estimated and the CT measured renal depth, and the correlation coefficient was calculated. The agreement between estimated and the CT measured renal depth was evaluated with Bland-Altman analysis reporting the bias (average of the differences between estimated and the CT measured renal depth), SD, and the 95% limits of agreement (average of the differences between estimated and the CT measured renal depth ± 1.96 × SD). Paired *t* test was performed between estimated and the CT measured renal depth.

## 3 Results

### 3.1 Correlation analysis

**Figure 2:**
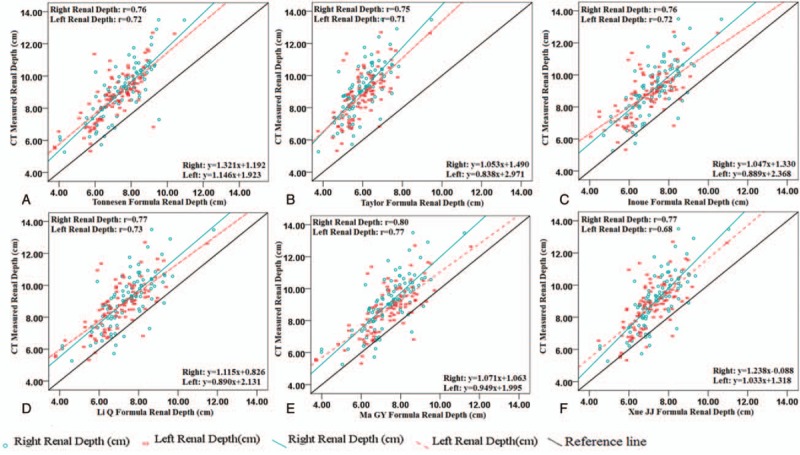
Relationship between estimated and the CT measured renal depth. The *P* values of all the *r* values were <.01.

There was a strong and significant correlation between estimated and CT measured renal depth, but Ma GY formula is better than the other 4 formulas; the correlation coefficients were 0.80 (*P* < .01) for right renal and 0.77 (*P* < .01) for left renal, respectively (Fig. 2). We found the scatter of the data about the regression line was substantial and different for both kidneys for the various formulas used (Fig. 2). While right renal depth was better than left renal depth, Li Q formula and Ma GY formula had better performance than other 4 formulas.

### 3.2 Bland-Altman analysis and paired *t* test

**Figure 3:**
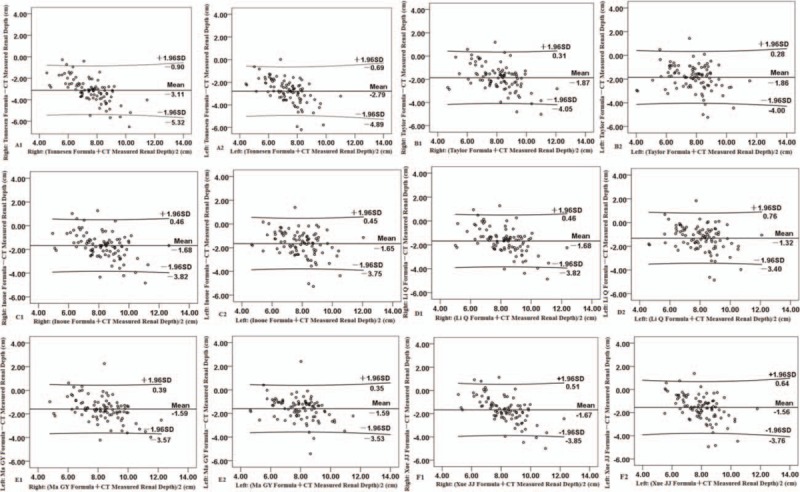
Bland-Altman analysis between estimated and the CT measured renal depth.

Estimated renal depths were lower than the CT measured renal depth. Based on the scatter plots in Figure 3, we found 6 formulas tended to underestimate renal depth for both kidneys. The scatter did not appear to be random but dependent on the depth as determined from the CT images. The error increased as CT measured renal depth increased. The renal depth derived from Tonnesen formula was significantly lower than the CT measured renal depth (Fig. 3, A1 and A2). The agreement between the estimated renal depth derived from Tonnesen formula and the CT measured renal depth was the worst, with the mean difference of (right: −3.11 ± 1.13 cm; left: −2.79 ± 1.07 cm). The correlation coefficients of Taylor formula were worse than Tonnesen formula (Fig. 2, A and B), but the agreement of Taylor formula was better, the deviation was −1.86 cm (Fig. 3, B1 and B2). The correlation coefficients of Inoue formula and Tonnesen formula were the same (Fig. 2, B and C), while the agreement of Inoue formula was much better than Tonnesen formula, the deviation was −1.66 cm (Fig. 3, C1 and C2). The agreement between the estimated renal depth derived from Li Q formula and Ma GY formula and the CT measured renal depth was the best (Fig. 3, D1–E2), with the mean difference of right: −1.68 ± 1.09 cm; left: −1.32 ± 1.06 cm and right: −1.59 ± 1.01 cm; left: −1.59 ± 0.99 cm, respectively. Although Li Q formula and Ma GY formula were better than the other 4 formulas, the greatest error between Li Q formula and Ma GY formula and the CT measured renal depth was up to −4.83 cm. Paired *t* test showed that there was significant difference between estimated and CT measured renal depth (Table 2).

**Table 2:**
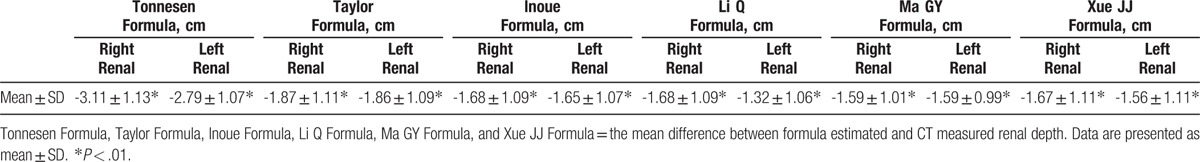
Mean difference between estimated and CT measured renal depth (paired *t* test).

## 4 Discussion

The renal depth is important in determining the attenuation coefficient used to calculate kidney function from scintigraphic scans.^[4]^ Most HSK patients have abnormal kidney rotation and fusion of the kidneys at the lower poles to form an isthmus that usually lies anterior to the great vessels at the level of the third to fifth lumbar vertebra creating a U-shape.^[12]^ Accordingly, the renal depth differs from the normal kidney. Therefore, use of existing formulas for estimating the renal depth in HSK patients will lead to errors, that is, when ΔX = 1 cm, GFR = 100 mL/min, ΔGFR/GFR = 17.65%.^[4]^ Previous study^[2]^ found that GFR measured by ^99m^Tc-DTPA renal dynamic imaging is significantly lower than estimated GFR which was estimated by the Chronic Kidney Disease Epidemiology Collaboration (CKD-EPI) equation in HSK patients. We postulated that some of this error could be explained by inaccuracies of estimated renal depth.

Correction for soft-tissue attenuation is necessary to quantitate renal accumulation, and an estimate of renal depth is commonly used for attenuation correction. The accuracy of a camera-based method depends on the accuracy of estimated renal depth. In this study, we estimated the accuracy of the existing 6 renal depth estimation formulas in HSK. The results showed that all the existing renal depth estimation formulas performed poorly and trended toward underestimated HSK's renal depth. When Tonnesen formula is used to estimate HSK's renal depth, the deviation of GFR will be up to approximately 54%. Although Li Q formula and Ma GY formula were better than the other 4 formulas, the greatest deviation of GFR was up to approximately 87% which was unacceptable. It is very important to accurately estimate the renal depth of HSK when using Gates method for the determination of GFR. To provide reliable and accurate renal depth, a new formula for HSK needs to be developed and validated.

Based on existing data, we have developed a new formula to estimate the renal depth in HSK patients. But the new formula was not satisfactory. In stepwise regression equations derived process, the contribution of the same variable was different in left and right renal depth equation. We speculate that may be the sample size is not big enough, so our next task is to collect more cases and set up a new formula then evaluate its accuracy.

## 5 Conclusion

All the existing renal depth estimation formulas do not apply to HSK. To provide reliable and accurate estimates of renal depth, we should develop a new formula to estimate the renal depth in HSK patients.

## References

[1] Weizer AZ Silverstein AD Auge BK Determining the incidence of horseshoe kidney from radiographic data at a single institution. J Urol 2003;170:1722–6. 14532762 10.1097/01.ju.0000092537.96414.4a

[2] Qi Y Hu P Xie Y Glomerular filtration rate measured by (99m) Tc-DTPA renal dynamic imaging is significantly lower than that estimated by the CKD-EPI equation in horseshoe kidney patients. Nephrology (Carlton) 2016;21:499–505. 26517584 10.1111/nep.12663 PMC5111751

[3] Inker LA Schmid CH Tighiouart H Estimating glomerular filtration rate from serum creatinine and cystatin C. N Engl J Med 2012;367:20–9. 22762315 10.1056/NEJMoa1114248 PMC4398023

[4] Ma GY Shao MZ Xu BX Establish new formulas for the calculation of renal depth in both children and adults. Clin Nucl Med 2015;40:e357–62. 26018708 10.1097/RLU.0000000000000808

[5] Gates GF Split renal function testing using Tc-99m DTPA. A rapid technique for determining differential glomerular filtration. Clin Nucl Med 1983;8:400–7. 6357589 10.1097/00003072-198309000-00003

[6] Madsen CJ Møller ML Zerahn B Determination of kidney function with 99mTc-DTPA renography using a dual-head camera. Nucl Med Commun 2013;34:322–7. 23426159 10.1097/MNM.0b013e32835f1620

[7] Tonnesen KH Mogensen P Wolf H Residual kidney function after unilateral nephrectomy. Pre- and postoperative estimation by renography and clearance measurements. Scand J Urol Nephrol 1976;10:130–3. 948721 10.3109/00365597609179672

[8] Taylor A Lewis C Giacometti A Improved formulas for the estimation of renal depth in adults. J Nucl Med 1993;34:1766–9. 8410296

[9] Inoue Y Yoshikawa K Suzuki T Attenuation correction in evaluating renal function in children and adults by a camera-based method. J Nucl Med 2000;41:823–9. 10809198

[10] Li Q Zhang CL Fu ZL Measuring kidney depth of Chinese people with kidney dynamic imaging. Chin J Med Imaging Technol 2007;23:288–91.

[11] Xue J Deng H Jia X Establishing a new formula for estimating renal depth in a Chinese adult population. Medicine (Baltimore) 2017;96:e5940. 28151880 10.1097/MD.0000000000005940 PMC5293443

[12] Natsis K Piagkou M Skotsimara A Horseshoe kidney: a review of anatomy and pathology. Surg Radiol Anat 2014;36:517–26. 24178305 10.1007/s00276-013-1229-7

